# Changes in distribution of waterbirds following prolonged drought reflect habitat availability in coastal and inland regions

**DOI:** 10.1002/ece3.2091

**Published:** 2016-08-30

**Authors:** Li Wen, Neil Saintilan, Julian R. W. Reid, Matthew J. Colloff

**Affiliations:** ^1^ Water, Wetlands and Coastal ScienceNew South Wales Office of Environment and Heritage59‐61 Goulburn StreetSydneyNSW2000Australia; ^2^ Department of Environmental SciencesMacquarie UniversitySydneyNSW2019Australia; ^3^ The Fenner School of Environment and SocietyAustralian National UniversityCanberraACT2601Australia; ^4^ CSIRO Land and WaterGPO Box 1700CanberraACT2601Australia

**Keywords:** Climate change, coasts, Maxent, nomadic waterbird, wetland management

## Abstract

Provision of suitable habitat for waterbirds is a major challenge for environmental managers in arid and semiarid regions with high spatial and temporal variability in rainfall. It is understood in broad terms that to survive waterbirds must move according to phases of wet–dry cycles, with coastal habitats providing drought refugia and inland wetlands used during the wet phase. However, both inland and coastal wetlands are subject to major anthropogenic pressures, and the various species of waterbird may have particular habitat requirements and respond individualistically to spatiotemporal variations in resource distribution. A better understanding of the relationships between occurrence of waterbirds and habitat condition under changing climatic conditions and anthropogenic pressures will help clarify patterns of habitat use and the targeting of investments in conservation. We provide the first predictive models of habitat availability between wet and dry phases for six widely distributed waterbird species at a large spatial scale. We first test the broad hypothesis that waterbirds are largely confined to coastal regions during a dry phase. We then examine the contrasting results among the six species, which support other hypotheses erected on the basis of their ecological characteristics. There were large increases in area of suitable habitat in inland regions in the wet year compared with the dry year for all species, ranging from 4.14% for Australian White Ibis to 31.73% for Eurasian Coot. With over half of the suitable habitat for three of the six species was located in coastal zones during drought, our study highlights the need to identify and conserve coastal drought refuges. Monitoring of changes in extent and condition of wetlands, combined with distribution modeling of waterbirds, will help support improvements in the conservation and management of waterbirds into the future.

## Introduction

Providing effective means for conservation of nomadic and migratory species is one of the more intractable problems facing environmental managers. The provision of networks of habitats and appropriate conditions that provide resources for feeding and breeding across connected landscapes is challenging in the context of rapidly expanding human populations and their environmental footprint (Woinarski et al. 1992). Oftentimes, the challenge is compounded by a lack of good information on the movement pathways of the species concerned.

Birds maintain the longest migratory distances of all terrestrial species and provide a good illustration of the challenges in identifying and maintaining biologically connected habitats. Migration flyways are threatened globally, and bird numbers in the East Asian–Australasian flyway have declined substantially in recent decades, due in part to reclamation of stopover habitat including the shores of the Yellow Sea (Moores 2006; Rogers et al. 2006). Summer habitat on the Australian continent has also been compromised by reclamation and water resource development, such as that leading to the salinization of the southern Coorong lagoon (Wainwright and Christie 2008; Paton et al. 2009). In some ways, nomadic bird movements pose even greater challengers, both to scientific understanding and in the provision and protection of critical habitat resources, given their apparent spatiotemporal unpredictability (Halse et al. 1998; Roshier et al. 2001a, 2001b; Padgham 2011).

**Figure 1:**
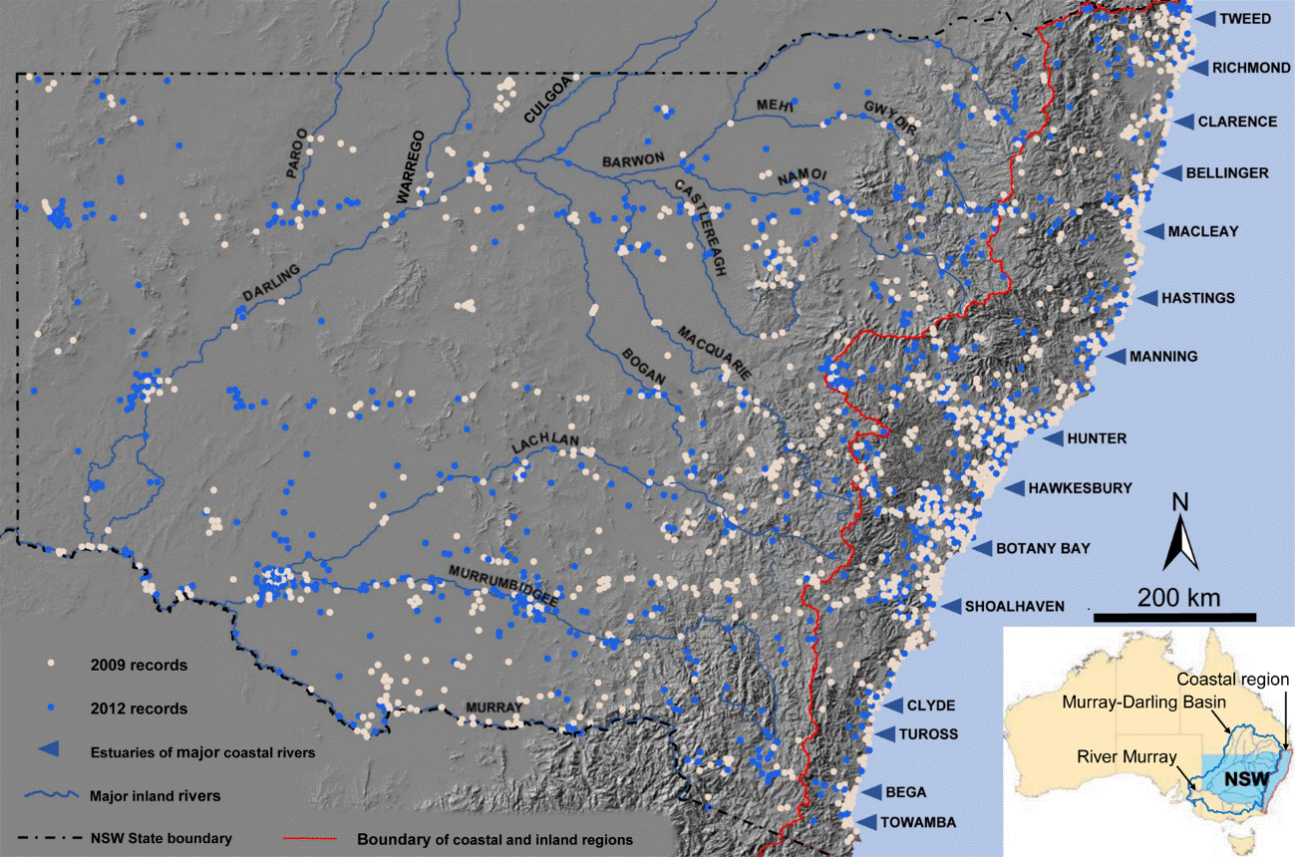
Map of study area, New South Wales, Australia. The waterbird sighting records in both 2009 (white) and 2012 (blue) were relatively widespread over the entire state. The hill shading derived from the 30 m digital elevation model shows the Great Dividing Range; the red line separates the coastal region (where rivers flow east into the Pacific Ocean) from the inland region, where rivers flow in a westerly direction toward the Southern Ocean via the single channel of the lower River Murray (inset).

Our study focusses on the habitat requirements of nomadic waterbirds on the Australian continent in relation to fluctuating climatic conditions. The rivers that flow west from the Great Dividing Range (Fig. 1) exhibit high variability in flows, a characteristic of rivers in arid and semiarid environments worldwide (Puckridge et al. 1998; Roshier et al. 2001a). The flooding of ephemeral wetlands releases nutrients and carbon, allowing for “boom” and “bust” cycles of resource availability for waterbirds (Kingsford et al. 1999; Baldwin et al. 2013). Under these conditions, wetlands may stay inundated for sufficient time to promote large breeding responses (Lawler and Briggs 1991; Morton et al. 1993; Kingsford et al. 1999). However, the spatial and temporal distribution of inland flooding is sporadic. Flows in the tributaries of the Darling River in the northern Murray–Darling Basin are influenced by intraseasonal variability of climate drivers that affect the southern penetration of tropical lows, such as the Julian–Madden Oscillation (Thoms et al. 2007; Murphy and Timbal 2008). The Murray and Murrumbidgee rivers of the southern basin supply large and more reliable flows than the northern tributaries. Here, as well as in the north, phases of the El Niño‐Southern Oscillation (Allen 1988; Verdon et al. 2004) and the Indian Ocean Dipole (Ummenhofer et al. 2009) exert strong influences on the timing and duration of droughts. Consequently, droughts may endure for several years then break suddenly in some (but not all) inland catchments. By adapting to the Australian landscape, waterbirds have developed the capacity to respond opportunistically to flooding across the mosaic of wetland landscapes of the interior in response to stochastic inundation events (Roshier et al. 2001b).

Anecdotal evidence has for some time suggested that waterbirds transit between flooded inland habitats during wet periods and coastal refuges when conditions are dry. Ibis and egrets nesting in the Macquarie Marshes in central New South Wales have been observed moving to the north coast during dry periods (McKilligan 1975; Geering et al. 1998). When Lake Eyre in central Australia was in flood in the mid‐1970s, pelicans were rarely seen in the coastal city of Brisbane, but became numerous after conditions in the interior had dried (Woodall 1985). In Toowoomba, an area with relatively high rainfall in Queensland (annual rainfall 724.3 mm, BOM, 2015), Straw‐necked Ibis populations in the 1970s reflected climatic conditions, with large numbers in the dry winter of 1970 and low numbers in the exceptionally wet winter of 1974 (McKilligan 1975). Drying conditions in central Australia appear to have, at various times, prompted the dispersal of Grey Teal to New Zealand and Papua New Guinea (Frith 1982), while Chambers and Loyn (2006) found that this species' abundance at a coastal refuge in Victoria correlated positively with streamflow in the Murray–Darling Basin 15–18 months beforehand.

An understanding of the circumstances under which waterbirds of different guilds use inland and coastal habitats is important in order to interpret population occurrence and trends. Water resource development and diversions for irrigation from the rivers of the Murray–Darling Basin have resulted in major changes in frequency, duration and extent of inundation of floodplain wetlands that historically have provided important habitat for waterbirds (Sims et al. 2012), and these trends are likely to continue with projections of a drying climate to 2100 across the region (Saintilan and Rogers 2013). For example, reduced flow in the lower Murrumbidgee, the largest tributary of the southern basin, led to an estimated 90% decline in waterbird numbers between 1983 and 2001 (Kingsford and Thomas 2004). A similar decline, between 1983 and 1993, was reported for the Macquarie Marshes (Kingsford and Thomas 1995). Impacts of development have also been severe in higher rainfall headwater regions (Brock et al. 1999) and on coastal wetlands in New South Wales (Goodrich 1970; Williams and Watford 1997), both regions providing important drought refugia (White 1987), but for which information on long‐term trends in waterbird populations is scarce. Identification of population trends is confounded by high interannual variability associated with nomadic movements driven by climate fluctuation (Kingsford et al. 2011; Colloff et al. 2015). A better understanding of the relationships between occurrence of waterbirds and habitat quality under changing climatic conditions would not only clarify trends but also help target investments in conservation.

In this manuscript, we provide the first predictive models of habitat availability for waterbirds between wet and dry climatic phases. We examine changes in waterbird distribution in relation to habitat availability during and after prolonged and severe drought for six species of abundant and widely distributed Australian waterbirds with contrasting life‐history characteristics and habitat requirements: Grey Teal *Anas gracilis*, Australian Wood Duck *Chenonetta jubata*, Eurasian Coot *Fulica atra*, Little Black Cormorant *Phalacrocorax sulcirostris*, Australian White Ibis *Threskiornis molucca,* and Masked Lapwing *Vanellus miles*. A summary of life‐history characteristics, movement strategies and broad habitat and dietary preferences of these six species (Table 1) allowed us to construct some expectations regarding the changes in waterbird distribution patterns. In particular, we tested the following hypotheses underlying the well‐documented phenomenon whereby waterbird populations increase rapidly in inland Australia after the break of long‐term drought events (Scott 1997):

**Table 1:** Life histories, broad habitat preferences, and movement strategies of the six species of waterbirds in this study, based on data summarized by Marchant and Higgins (1990, 1993) and Rogers and Ralph (2010), and with functional group membership from Roshier et al. (2002) and species associations from Fjeldså (1985)

Species	Macrohabitat zones	Foraging habitat	Broad diet	Functional group	Fjeldså association	Mobility	Dispersion in drought
Grey Teal	w/s	Aquatic	Om, Zoop	Dabbling duck	4	H	F
Australian Wood Duck	w/s	Terrestrial	Veg	Grazing waterfowl	5	L	D*
Eurasian Coot	w/s	Aquatic	Om, Veg	Deep water forager	3	H	F
Little Black Cormorant	IR, C	Aquatic	Fish	Fish eater	1	H	F
Australian White Ibis	(IR), C	Generalist	Inv	Large wader	1	L	D*
Masked Lapwing	w/s, C	Shoreline	Inv	Shoreline forager	5	L	D

**Figure 2:**
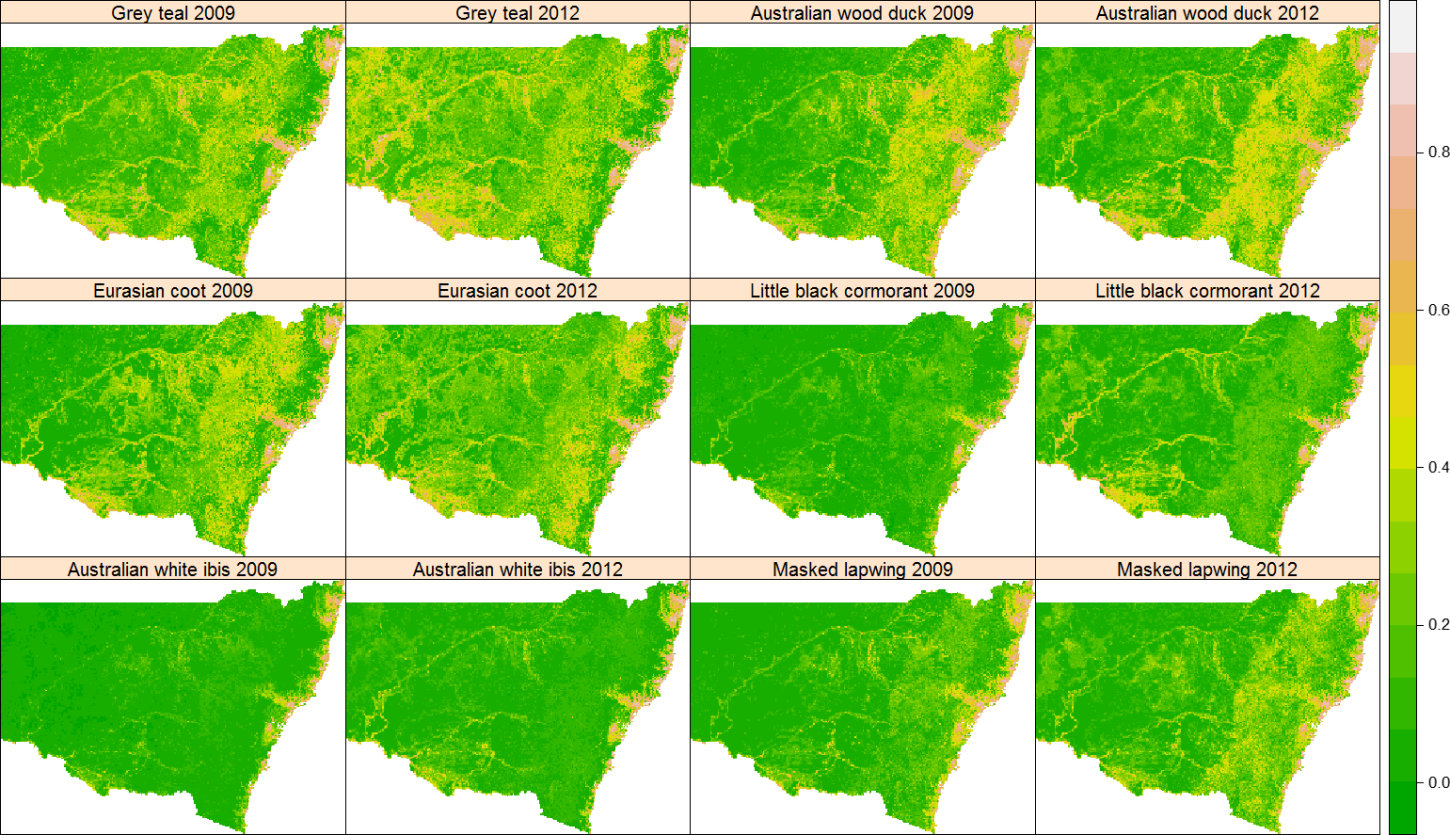
Predicted maps of relative habitat suitability, based on probability of occurrence, of six waterbird species in 2009 and 2012 using topographic and NDVI predictor variables.

*Macrohabitat zones*: w/s, widespread; IR, Inland Rivers; C, coastal; (), semi‐dependent. *Broad diet*: Om, omnivore; Zoop, zooplankton; Veg, plant foods; Inv, invertebrates. *Fjeldså association*: five broad interspecific associations recognized by cluster analysis based on waterbird inventories across 271 NSW wetlands (Fjeldså 1985; Figs 1 and 2 therein). *Mobility*: a dichotomous classification representing higher mobility (H), including a higher proportion of populations likely to move, and lower mobility (L). *Dispersion*: F, focussed (large flocks in non‐breeding situations and drought); D, dispersed, *while these species also form large flocks at times, they remain widely dispersed in higher rainfall and coastal regions during drought.

1. Waterbirds, especially highly mobile, dispersive species, such as Grey Teal, Eurasian Coot, and Little Black Cormorant (Table 1), would use wetlands in coastal catchments as refuges during dry periods, and therefore, the proportion of suitable habitat in the coastal zone would be larger in the dry phase than in the wet phase. In addition, in association with improved productivity during the wet phase, we expected there would be a higher occurrence of most or all waterbird species during the wet phase than during the dry phase;
2. During wet years, when productivity may not be a limiting factor, the predictor variable we used to represent productivity‐Normalised Difference Vegetation Index (NDVI) in this study‐would make a lower contribution to the predictive power of the model than during dry years;
3. The similarity in distribution between dry and wet years should be higher for those species with low mobility such as Australian Wood Duck and Masked Lapwing and lower for species with higher mobility such as Grey Teal and Little Black Cormorant; and
4. The similarity in distribution among species would be higher during the dry period, especially for the inland regions where prevailing poor wetland conditions force waterbirds to concentrate in drought refugia. This pattern is likely to be more obvious when comparing the highly mobile species such as Grey Teal and the species which are thought to generally move shorter distances such as Australian Wood Duck.

## Methods

### Study area

We modeled the distribution of the six waterbird species over all of New South Wales and the Australia Capital Territory (ca. 810,000 km^2^). The study area is separated into inland and coastal regions by a series of highlands and plateaus termed the Great Dividing Range, which runs parallel with the east coast (Fig. 1). The main rivers rise in the Great Dividing Range, with the coastal rivers flowing eastwards to the Pacific Ocean, and the inland rivers flowing westwards and eventually combining with the Murray and Darling rivers which flow to the Southern Ocean via the lower River Murray in South Australia. The coastal rivers are relatively short and tend to lack major floodplains, though some terminate in extensive estuaries and lakes. Almost all the major inland rivers are regulated with weirs and dams and have extensive lowland floodplains and wetlands that provide important habitat for waterbirds.

### Waterbirds occurrence data

We accessed a large database of waterbird sight records (occurrence) from the Atlas of New South Wales Wildlife (www.bionet.nsw.gov.au), from observations made by scientists and staff of government agencies and universities. When supported by environmental data, these records provide insights into responses of waterbirds to hydrological, climatic, and other drivers (Wen et al. 2011). We obtained data on sightings of the six waterbird species in 2009, the last year of the Millennium Drought (1997–2009), and in 2012, following very high rainfall during consecutive, strong La Niña years in 2010 and 2011. We excluded records with a spatial accuracy of >100 m. In addition, data from the Aerial Waterbird Survey of Eastern Australia (AWSEA) were also included. The AWSEA counts waterbirds annually since 1983 at large wetlands along 12 latitudinal transects extending from 20°30′S in Queensland to 38°30′S at the southern tip of the continent (Kingsford et al. 2011). Although the AWSEA is a standardized survey which produces both presence and absence data for surveyed wetlands therefore has the potential to produce more accurate distribution models than the ones produced by presence‐only data (Guillera‐Arroita et al. 2015), it is not suitable for systematic waterbird distribution study due to the small number of data records (e.g., there are only four records for Australian White Ibis, Eurasian Coot, and Masked Lapwing for 2009).

### Predictor variables

#### NDVI predictor variables

We used the mean and coefficient of variation (CV) of monthly MODIS NDVI for the entire state of New South Wales in 2009 and 2012. NDVI, an index of vegetation greenness or vigor and a surrogate for primary productivity and biomass (Goward and Dye 1987; Cho et al. 2007), is used for predictor variables because it indicates resource availability. It has been used previously to explain species distribution and abundance (Skidmore et al. 2003; Evans et al. 2005), especially in highly fragmented landscapes (Wen et al. 2015). The CV of NDVI relates to seasonal variation in primary productivity and is a surrogate of habitat quality, modified by local climate and soil nutrient status (Wiegand et al. 2008).

#### Topographic predictor variables

Although indirect, topographic variables are important because of their influence on local climate (Moore et al. 1991), vegetation (Franklin 1995), and water availability. Using the 1‐sec Shuttle Radar Topographic Mission Digital Elevation MODEL (STRM DEM) (http://www.ga.gov.au/), we calculated the topographic wetness index (TWI) for the entire study area. TWI accounts for elevation, direction of streamflow, and flow accumulation (Moore et al. 1993). We also included elevation as a candidate predictor variable. Because river flows are critical drivers of the ecological function of wetlands (Nilsson and Svedmark 2002), we included river density, calculated from channel patterns in the STRM DEM. These three predictors are constant in 2009 and 2012.

Waterbirds distribution in 2009 and 2012 was modeled separately using the occurrence data and NDVI‐derived variables in the corresponding year and the static topographic predictors.

### Distribution modeling

#### Model building

We used the software package Maxent version 3.3.3k (Phillips et al. 2006; Phillips and Dudik 2008) to predict the distribution of each species from the occurrence data. With its root in information theory, Maxent is a machine‐learning approach to predictive niche modeling that quantifies the association between the occurrence of a species and the site's environmental conditions (Phillips et al. 2006). Using a set of features (i.e., transformations of the original predictor variables include linear, product and quadratic), Maxent iteratively minimizes the relative entropy between the probability density at the presence sites and the probability density of the landscape to find a model that can best differentiate presences from background locations (Phillips and Dudik 2008). Consequently, like other ecological niche models, Maxent allows assessing the relative suitability of habitat in geographic areas not sampled or occupied by a certain species (Warren and Seifert 2011). Maxent has been successfully validated (Elith et al. 2006) and is one of the most popular tools for species distribution and environmental niche modeling (Merow et al. 2013).

We set Maxent to randomly select 10,000 background points (~1% of the total pixels). Because the occurrence dataset included both systematic survey (i.e., AWSEA) and more site‐focused studies and incidental sightings, it is assumed that the samples were sufficiently unbiased from the model domain (Fig. 1) therefore not to require a bias map for the analysis. We used the “auto features” option with the recommended default values of 10^−5^ for the convergence threshold, 500 for the maximum number of iterations, and 1 for the regularization value. Because the correlation between the four predictors we used was low (the pairwise Pearson coefficient ranges from 0.14 to 0.38), there is little issue of multicollinearity; thus, the risk of overfitting is low. For all species, a random selection of 75% of the occurrence points was used for model training and the remaining 25% for model testing. Using bootstrap resampling, the modeling process was replicated 30 times to test the model performance and to measure the amount of variability in the model. The average of the 30 logistic outputs, which assign a probability of occurrence of a given species to each cell in the study region, was presented and used for further analysis.

To facilitate the comparison of distribution between dry and wet years, we further classified the maps of probability of occurrence into a binary map of suitable/unsuitable habitats using the equal training sensitivity–specificity thresholds. The sensitivity–specificity equality approach performs as well or better than 11 other methods for selecting threshold values (Liu et al. 2005).

#### Model evaluation

We used receiver operating characteristic analysis (ROC) to assess model performance. Sensitivity (true positives) were plotted against 1 minus specificity (false positives) for a range of threshold values, with the area under the curve (AUC) indicating the capacity of the model to discriminate presence from absence. A random prediction gives an AUC value of 0.5, whereas a perfect prediction gives the maximum AUC of 1.0 (Fielding and Bell 1997); values >0.70 are considered acceptable (Lemeshow and Hosmer 1982) and values >0.75 are deemed suitable for conservation planning (Pearce and Ferrier 2000; but see Lobo et al. 2008). AUC has increasingly been used for evaluation of models of species distributions (Elith 2000; Vanagas 2004) and has the advantage of providing a single measure of model performance independent of the choice of threshold (Phillips et al. 2006).

#### Comparison of maps of predicted distribution

We calculated *D*_SDM_, the *D* index of Schoener (1968), which is a classical and reliable measure of niche overlap (Rödder and Engler 2011) widely used in ecological studies and SDM applications in particular, to quantify the similarity between SDMs in wet and drought years. *D*_SDM_ ranges from 0 (models have no overlap) to 1 (models are identical) and are derived from the difference in probability distributions over space produced between two SDMs. We calculated the *D*_SDM_ for both the occurrence probability maps and binary maps of suitable/unsuitable habitats using the equal training sensitivity–specificity thresholds.

We also identified the areas of significant change between dry and wet years in terms of species occurrence probability. The grid‐specific significance of the pairwise differences (between the two predictions for 2009 and 2012) was calculated relative to the mean and variance across all grids over the model domain (Januchowski et al. 2010; Bateman et al. 2012). The resultant rasters representing the individual significance values were reclassified into three classes: areas where the prediction in 2009 indicated significantly more suitable habitat than in 2012 (SD ≥ 0.975), areas where there was significantly less suitable habitat in 2009 than 2012 (SD ≤ 0.025), and areas where there was no significant difference between 2009 and 2012.

We used R 3.0.2 (R Development Core Team, 2013) for all statistical analyses, the package dismo 0.9‐3 (Hijmans et al. 2013) for niche similarity test, SDMTools 1.1‐20 (VanDerWal et al. 2014) for patch analysis and prediction comparisons, and Raster 2.1‐25 (Hijmans et al. 2013) for grid data transformation and visualization.

## Results

### Performance of models

The sample size of each of the six waterbird species was moderately large, ranging from 264 (Eurasian Coot in 2009) to 707 (Australian Wood Duck in 2012, Table 2). For all fitted models, the AUC values were significant greater than random (AUC >0.5) according to the ties‐corrected Mann–Whitney *U*‐test (*P *<* *0.001 for all cases), indicating adequate fitting. Also, difference between training and testing AUCs was small (ranged from 0.01 for Australian White Ibis in 2009 to 0.04 for Eurasian Coot in 2009; Table 2), and the standard deviation of model AUC based on 30 bootstrap runs was small for all species, suggesting little over‐fitting of model predictions, and the Maxent algorithms captured variations in environmental predictors over the occurrence points.

**Table 2:** The performance of the fitted species distribution models. AUC Standard Deviation is based on 30 bootstrapped runs

Species/year	Training samples	Training AUC	Testing samples	Testing AUC	AUC Standard deviation
Dry year (2009)					
Grey Teal	362	0.87	120	0.84	0.02
Australian Wood Duck	663	0.89	221	0.87	0.01
Eurasian Coot	252	0.91	83	0.87	0.02
Little Black Cormorant	306	0.95	102	0.93	0.01
Australian White Ibis	315	0.95	104	0.93	0.01
Masked Lapwing	587	0.94	195	0.92	0.01
Wet year (2012)					
Grey Teal	439	0.85	146	0.82	0.02
Australian Wood Duck	707	0.87	235	0.85	0.01
Eurasian Coot	329	0.88	109	0.85	0.02
Little Black Cormorant	456	0.91	152	0.89	0.01
Australian White Ibis	318	0.92	106	0.89	0.02
Masked Lapwing	576	0.91	192	0.89	0.01

All models had robust performance. AUCs (both training and testing) were slightly higher (by 0.02–0.04) for 2009 models of all species than 2012 (Table 2). However, differences were not significant (Mann–Whitney *U*‐tests on 30 AUC values from bootstrapping runs).

### Waterbird distributions and habitat suitability in dry and wet years

Habitat suitability based on the probability of occurrence was higher in coastal regions than inland for all species, regardless of whether the year was dry or wet (Fig. 2). The expansion of suitable habitat in inland regions in the wet year of 2012 was clear for all species, but particularly for Grey Teal in northern, western, and south‐western regions; Australian Wood Duck in southern regions; Eurasian Coot, Little Black Cormorant, and, to a lesser extent, Australian White Ibis along the Darling, Murray, Lachlan and Murrumbidgee rivers. By way of contrast, habitat suitability of Masked Lapwing increased mostly in regions east of western foothills of the Great Dividing Range.

**Figure 3:**
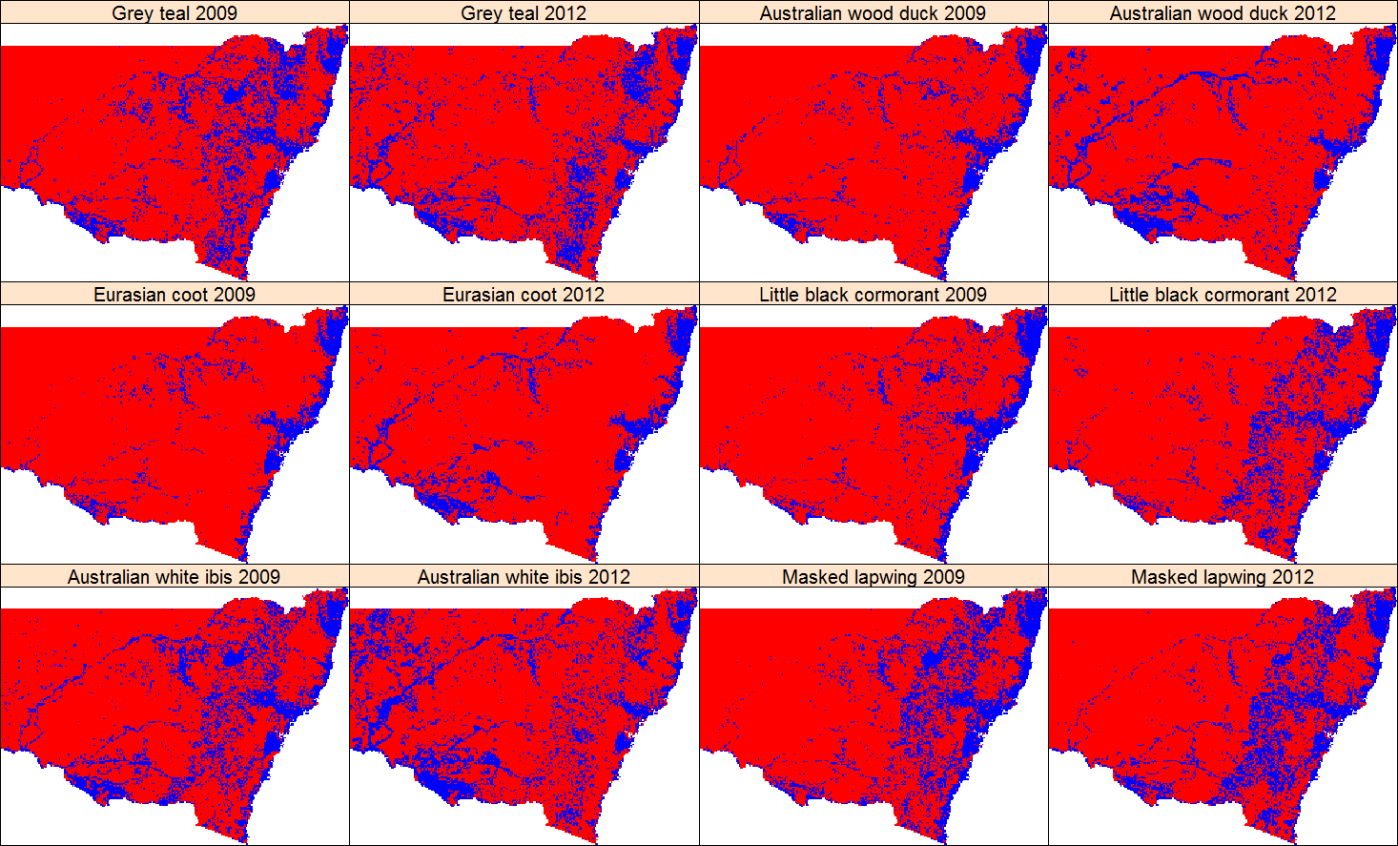
The predicted distribution of six waterbird species in 2009 and 2012 based on binary habitat suitability, using equal training sensitivity–specificity thresholds. Blue = 1, suitable; and Red = 0, not suitable.

The patterns derived from probability of occurrence were generally reflected in the results of the predicted distribution patterns, which showed all six waterbird species had more extensive distributions in the wet year of 2012 than in the dry year of 2009 (Fig. 3). All waterbird species except Australian Wood Duck showed marked expansion in regions west and north‐west of the Darling River compared with the dry year 2009.

From the maps of binary habitat suitability (Fig. 3), we calculated changes in area of suitable habitat between 2009 and 2012 (Table 3). The increase in total area of suitable habitat in the wet year (2012) ranged from 4.14% for Australian White Ibis to 31.73% for the Eurasian Coot, a deep water forager. In the inland regions, there were increases in area of suitable habitat in the wet year of 2012 for all species between 22.07% for Australian White Ibis and 77.86% for Masked Lapwing, a shoreline forager. Coincident with the increases in the inland region, the probability of occurrence in relation to available coastal habitat decreased in the coastal catchments for all species except Eurasian Coot, with the largest decrease for Masked Lapwing (1.02 million ha or 19.15% reduction). In the inland regions, there were some areas that were not suitable habitats in 2012 but which were suitable in 2009. These areas were generally headwaters including the Macquarie–Castlereagh and upper Namoi catchments (Fig. 3). No two species showed the same pattern of increase across the inland region in the wet phase; rather, each species exhibited an individualistic response to increased wetland opportunities.

**Table 3:** Modeled waterbird habitat area (millions of hectares) in dry (2009) and wet (2012) years based on binary maps of probability of occurrence (Fig. 3) for coastal and inland regions and the entire New South Wales

	NSW			Inland			Coastal		
	2009	2012	± (%)	2009	2012	± (%)	2009	2012	± (%)
Grey Teal									
Total area (million ha)	12.01	14.56	21.20	7.61	11.13	46.27	4.40	3.42	−22.19
Mean patch area (ha)	77.40	79.26	2.40	58.99	70.19	18.98	167.26	134.88	−19.36
Landscape proportion (%)	15	18	21.20	11	16	46.27	34	27	−22.19
Total core area (million ha)	3.50	4.53	29.27	1.47	2.95	100.24	2.03	1.57	−22.28
Core proportion (%)	4	6	29.27	2	4	100.24	16	12	−22.28
Australian Wood Duck									
Total area (million ha)	13.57	17.32	27.56	7.72	11.76	52.37	5.85	5.54	−5.17
Mean patch area (ha)	105.10	125.66	19.56	76.09	112.06	47.26	209.89	167.69	−20.10
Landscape proportion (%)	17	22	27.56	11	17	52.37	45	43	−5.17
Total core area (million ha)	4.63	6.28	35.57	1.60	3.45	115.10	3.02	2.82	−6.68
Core proportion (%)	6	8	35.57	2	5	115.10	24	22	−6.68
Eurasian Coot									
Total area (million ha)	10.03	13.21	31.73	6.20	9.19	48.26	3.83	4.02	4.91
Mean patch area (ha)	72.52	83.84	15.61	57.26	69.76	21.83	126.49	153.57	21.41
Landscape proportion (%)	12	16	31.73	9	14	48.26	30	31	4.91
Total core area (million ha)	3.07	3.84	24.77	1.29	1.95	51.00	1.78	1.87	5.37
Core proportion (%)	4	5	24.77	2	3	51.00	14	15	5.37
Little Black Cormorant									
Total area (million ha)	8.99	10.14	12.77	4.13	6.67	61.77	4.86	3.46	−28.82
Mean patch area (ha)	78.20	79.11	1.17	46.82	63.37	35.35	180.48	150.88	−16.40
Landscape proportion (%)	11	13	12.77	6	10	61.77	38	27	−28.82
Total core area (million ha)	3.75	3.99	6.45	1.12	2.04	81.72	2.62	1.95	−25.70
Core proportion (%)	5	5	6.45	2	3	81.72	20	15	−25.70
Australian White Ibis									
Total area (million ha)	6.85	7.13	4.14	3.19	3.89	22.07	3.66	3.23	−11.56
Mean patch area (ha)	60.66	53.59	−11.66	36.46	34.81	−4.51	143.55	151.29	5.40
Landscape proportion (%)	9	9	4.14	5	6	22.07	28	25	−11.56
Total core area (million ha)	2.22	2.42	9.04	0.51	0.82	59.80	1.71	1.60	−6.26
Core proportion (%)	3	3	9.04	1	1	59.80	13	12	−6.26
Masked Lapwing									
Total area (million ha)	9.57	11.83	23.65	4.22	7.50	77.86	5.34	4.32	−19.15
Mean patch area (ha)	73.84	74.74	1.21	41.37	61.23	48.00	193.23	120.32	−37.73
Landscape proportion (%)	12	15	23.65	6	11	77.86	42	34	−19.15
Total core area (million ha)	3.44	3.23	−6.16	0.72	1.40	93.97	2.72	1.82	−32.87
Core proportion (%)	0.04	0.04	−6.16	0.01	0.02	93.97	0.21	0.14	−32.87

**Figure 4:**
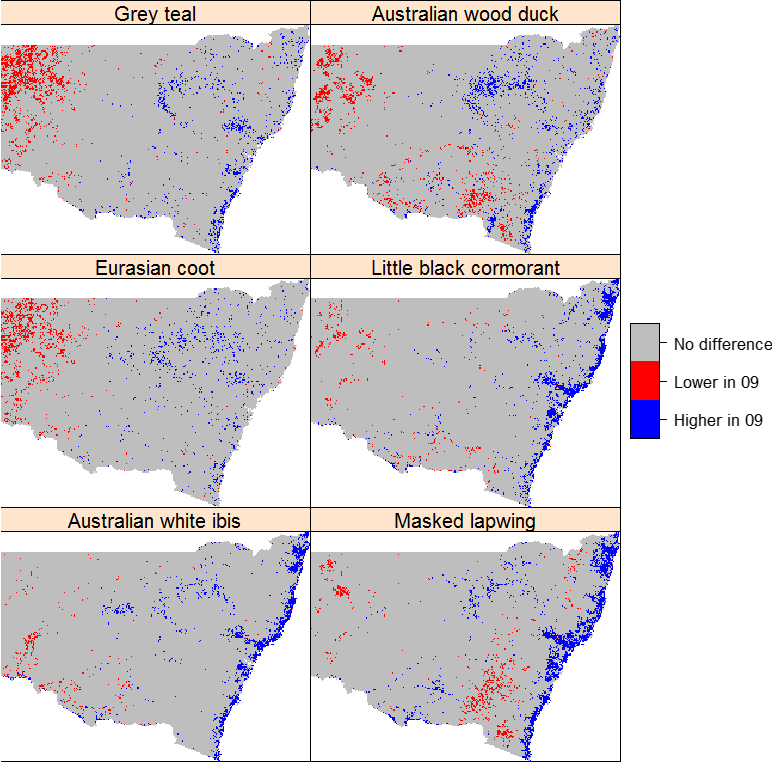
Comparison of pairwise differences relative to the mean and variance of all differences between Maxent predictions of occurrence probability of six waterbird species in 2009 (dry) and 2012 (wet). Blue = significantly higher probability in 2009; red = significantly lower probability in 2009.

The maps of areas of statistically significant change in habitat suitability based on Maxent predictions (Fig. 4) indicate no significant change in probability of waterbird occurrence between wet and dry years for over 80 million ha (all species, Table 4). However, this result should be interpreted with caution because a large part of the area with no change was not suitable for waterbirds in either wet or dry years (Figs 2 and 3).

**Table 4:** Changes (millions of hectares) in habitat suitability between dry (2009) and wet (2012) years based on maps of the logistic probability of occurrence of waterbirds from Maxent predictions (Fig. 4) for coastal and inland regions and for all of the study region (sum of coastal and inland regions). Improved = areas where probability of occurrence in 2009 is significantly higher (SD ≥ 0.975); decreased = areas where probability of occurrence in 2009 is significantly lower (SD ≤ 0.025)

Species	No change			Improved			Decreased		
	Coastal	Inland	NSW	Coastal	Inland	NSW	Coastal	Inland	NSW
Grey Teal	12.67	68.77	81.44	1.34	0.95	2.28	0.04	5.21	5.25
Australian Wood Duck	12.66	68.35	81.00	1.29	1.77	3.06	0.10	4.42	4.51
Eurasian Coot	13.35	69.11	82.46	0.63	1.37	2.01	0.06	4.33	4.40
Little Black Cormorant	11.12	71.29	82.41	2.91	0.79	3.70	0.01	4.25	4.27
Australian White Ibis	11.46	71.26	82.72	2.54	0.94	3.49	0.04	3.56	3.60
Masked Lapwing	10.24	70.24	80.47	3.74	1.12	4.86	0.07	3.17	3.24

For the entire study region, the areas where the suitability of waterbird habitat was significantly decreased in the dry year of 2009 ranged from 3.24 million ha for Masked Lapwing to 5.25 million ha for Grey Teal (Table 4), and the areas with increased suitability in 2009 ranged from 2.01 to 4.86 million ha. Regions where suitability was significantly higher in 2012 included the catchments of the lower Darling, the middle and lower Murrumbidgee and Lachlan rivers, and the catchments west of the Darling (Fig. 4). By contrast, the majority of the areas with significantly improved suitability in 2009 were located in headwaters and coastal catchments.

### Distribution difference between dry and wet years

For all species, the proportion of suitable habitat in the coastal zone was much higher during the dry phase (Tables 4 and 5). More than 50% of the 2009 modeled suitable habitat of Little Black Cormorant, Australian White Ibis and Masked Lapwing was in the coastal region. However, the proportions decreased considerably in the wet year of 2012. The reduction ranged from 15.07% for Australian White Ibis to 36.88% for Little Black Cormorant.

**Table 5:** Distribution similarity (Schoener's D) between dry (2009) and wet (2012) years at New South Wales, inland and coastal regions

Species	Mobility	Guild	NSW	Inland	Coast
Australian Wood Duck	L, D	Grazing waterflowl	0.692	0.634	0.801
Australian White Ibis	L, D	Larger wader	0.572	0.441	0.710
Eurasian Coot	H, F	Deep water forager	0.594	0.510	0.765
Masked Lapwing	L, D	Shoreline	0.611	0.504	0.761
Little Black Cormorant	H, F	Fish eater	0.586	0.497	0.729
Grey Teal	H, F	Dabbling duck	0.516	0.436	0.726

For the entire model domain, the overlap of waterbird distribution between wet and dry years was “high” for Masked Lapwing (0.611) and Australian Wood Duck Grey Teal (0.692), and “moderate” for other species (0.516–0.594, Table 5) according to the criteria of Rödder and Engler (2011). For the inland regions, only Australian Wood Duck had high niche overlap while Schoener's D was moderate for other species. By contrast, the overlapping index was high for all species in the coastal zone. Regardless of these minor variations between species in the two regions, a paired *t*‐test comparing the degree of spatial overlap across years in inland vs coastal regions among the six species was highly significant (*t*_5_ = −14.07, *P *<* *0.001), with all species having a much higher degree of between‐year overlap in coastal areas (0.73–0.80) than inland (0.44–0.69). That is, all species redistributed themselves to a much greater degree in inland regions when the opportunities arose in 2012.

### Distribution difference among species

The comparison of distribution among species for dry and wet years (Table 6) shows the similarity among distribution patterns was higher in the coastal region for both wet and years ranging from high to very high overlap, whereas the overlap was moderate for the majority of cases in inland area. In general, the distribution ranges were more similar during drought when the geographic ranges were contracted, and this is particularly true for the inland area where only in two occasions (of 15), the similarity was higher in wet year.

**Table 6:** Distribution similarity (Schoener's D) among waterbird species in New South Wales, inland and coastal regions for 2009 and 2012 (in parentheses)

	Australian White Ibis	Eurasian Coot	Masked Lapwing	Little Black Cormorant	Grey Teal
NSW					
Australian Wood Duck	0.58 (0.54)	0.60 (0.60)	0.63 (0.57)	0.76 (0.61)	0.65 (0.67)
Australian White Ibis		0.68 (0.60)	0.75 (0.57)	0.64 (0.61)	0.61 (0.49)
Eurasian Coot			0.72 (0.61)	0.74 (0.70)	0.69 (0.62)
Masked Lapwing				0.62 (0.64)	0.60 (0.56)
Little Black Cormorant					0.72 (0.61)
Inland					
Australian Wood Duck	0.46 (0.46)	0.51 (0.50)	0.56 (0.45)	0.66 (0.52)	0.55 (0.63)
Australian White Ibis		0.64 (0.55)	0.66 (0.41)	0.55 (0.54)	0.56 (0.40)
Eurasian Coot			0.59 (0.57)	0.63 (0.63)	0.63 (0.55)
Masked Lapwing				0.48 (0.59)	0.51 (0.49)
Little Black Cormorant					0.67 (0.57)
Coast					
Australian Wood Duck	0.74 (0.71)	0.74 (0.78)	0.74 (0.77)	0.87 (0.83)	0.84 (0.82)
Australian White Ibis		0.76 (0.72)	0.83 (0.82)	0.79 (0.80)	0.79 (0.78)
Eurasian Coot			0.87 (0.71)	0.85 (0.86)	0.83 (0.87)
Masked Lapwing				0.81 (0.78)	0.76 (0.79)
Little Black Cormorant					0.80 (0.76)

### Importance of predictor variables and response curves

We reported the relative importance of environmental predictor variables (Table 7) based on the permutation test because it partly corrects for bias and is an improvement on the standard percent contribution (Strobl et al. 2007). In all cases, the predictors representing the changes in productivity (i.e., mean and CV NDVI) had considerable contribution to model prediction power (Table 7). Furthermore, the contribution of these predictors was significant lower in the wetter 2012.

**Table 7:** The permutation importance of predictor variables, expressed as percentages, and change in importance of predictor variables in dry (2009) and wet (2012) years

Species	Elevation			CV of NDVI			Mean NDVI			River density			TWI			Prevalence		
	2009	2012	*P*	2009	2012	*P*	2009	2012	*P*	2009	2012	*P*	2009	2012	*P*	2009	2012	*P*
Grey Teal	23.88	27.68	0.01	19.59	13.05	0.00	28.05	26.68	0.39	17.91	17.06	0.44	10.57	15.52	0.00	0.12	0.19	0.00
Australian Wood Duck	19.27	13.70	0.00	20.84	18.99	0.00	36.34	26.83	0.00	17.93	31.72	0.00	5.61	8.76	0.00	0.12	0.16	0.00
Eurasian Coot	12.22	23.82	0.00	14.97	19.37	0.02	33.92	23.11	0.00	31.33	22.31	0.00	7.57	11.39	0.00	0.08	0.13	0.00
Little Black Cormorant	34.50	28.49	0.00	16.68	12.61	0.00	12.59	12.14	0.54	33.45	41.92	0.00	2.79	4.85	0.00	0.04	0.08	0.00
Australian White Ibis	42.23	37.63	0.03	9.74	10.56	0.26	21.82	16.40	0.05	23.49	29.54	0.98	2.72	5.86	0.00	0.02	0.04	0.00
Masked Lapwing	22.95	16.85	0.09	26.64	30.69	0.02	30.29	27.72	0.08	16.30	18.90	0.08	3.82	5.84	0.00	0.05	0.07	0.00

*P*‐values are for the *t*‐tests based on the 30 bootstrap runs.

The response curves of species occurrence probability to annual mean NDVI were distinctive between dry and wet years (Appendix 1). The occurrence probability was high at water areas (NDVI ≤ 0) at both years for all species except the Eurasian Coot in 2009. By contrast, the occurrence probability was low at areas with a high NDVI value, a feature of forested regions.

## Discussion

The geographic distribution range or ecological niche is often conceptualized as a fixed aspect of a species and treated as such for the purposes of conservation (Runge et al. 2016). Absence from known distribution, especially from designated reserves, often signals an alert for management action. However, many avian species, in particular waterbirds, are mobile, and their movements range from regular seasonal migrations to unpredictable movements shown by nomadic species (Roshier et al. 2008). These movements can lead to substantial temporary expansion and contraction of geographic distribution ranges and might reflect the adaption strategies of the nomadic waterbirds to the variable climatic conditions.

By linking occurrence with the corresponding environmental conditions at the time of observations, we modeled and compared the distribution of six nomadic waterbirds in dry and wet phases across New South Wales, Australia. Our results provided multiple lines of evidences suggesting that waterbird utilize the coastal zone as drought refugia.

### Responses to climate variability: utilizing coastal wetlands as refuges

**Figure 5:**
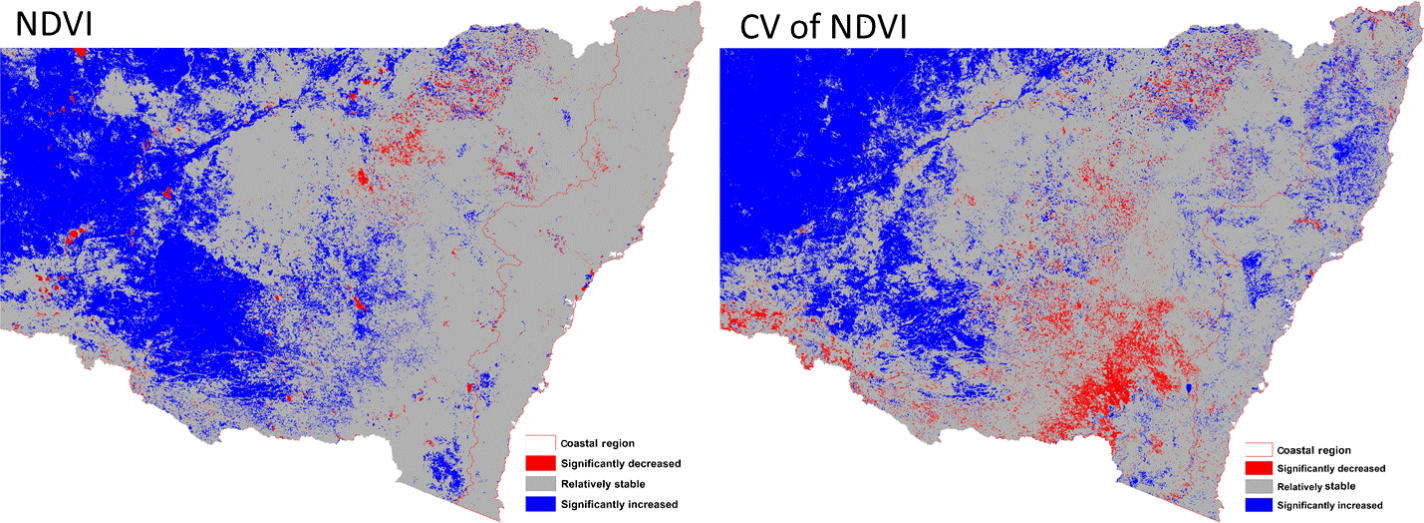
The changes in NDVI and coefficient of variation of NDVI between 2009 and 2012 for the New South Wales.

The increase of abundance and diversity of waterbirds following the break of drought is well documented in the arid/semiarid inland Australia (Scott 1997; Kingsford et al. 1999). In general, distributional range and abundance are positively correlated (Brown 1984). The large increase in suitable habitat for all waterbird guilds in the wet year of 2012 (ranging from 22.07% for Australian White Ibis to 77.86% for Masked Lapwing) confirmed this positive relationship. For all species, the coastal proportion of suitable habitat was much higher in 2009 implying the importance of coastal wetlands as waterbird refuges during dry periods as suggested in our first hypothesis. Moreover, the prevalence was significantly higher in 2012 than in 2009 suggesting that the overall higher land productivity (Fig. 5) facilitated the expansion of geographic distribution range to inland regions in wet phase. Furthermore, as suggested in our second hypothesis, the contribution of mean and seasonality of NDVI to the model was significantly lower in wet year than that in dry years implying that wet conditions enhanced land condition and homogenized its spatial variation as suitable habitat (Wen et al. 2012).

Coastal areas of the Australian continent have been recognized as an important dry‐season refuge for some time (Gentilli and Bekle 1983; Loyn et al. 1994). A study of waterbird utilization in one such refuge, Westernport Bay in Victoria, suggested a relationship between bird numbers and the Southern Oscillation Index, although this association was not as strong as with regional rainfall and streamflow data (Chambers and Loyn 2006). McKilligan (1975) attributed dry period use of coastal northern New South Wales by waterbirds to higher and more reliable rainfall and therefore a more reliable food supply. In our study area, the mean NDVI in 2009 was much higher in coastal areas (0.68 ± 0.12, spatial mean and SD) than in the inland region (0.34 ± 0.14, spatial mean and SD) implying the positive link between productivity and size of geographic range. However, in wet years, when large inland areas become suitable waterbird habitat with abundant food resources, demonstrated by the increase of NDVI (the spatial mean and SD of NDVI was 0.41 ± 0.13 and 0.34 ± 0.14 for 2012 and 2009), the relative habitat suitability and probability of occurrence in coastal regions decreased while those in inland areas increased. Sequentially, when applying a universal threshold to transform the initial probabilistic predictions (Fig. 2) into binary predictions (Fig. 3), the area classified as “suitable habitat” decreased in coastal regions as reported in Table 3.

### Responses of particular waterbird species to changes in habitat availability

The (dis)similarity of distribution between dry and wet years might reflect the life histories and movement strategies of the species. Species that interact with resources at broader scale, such as Grey Teal and Little Black Cormorant with high mobility, would be expected to have more divergent distributions between dry and wet years. Thus, their between‐year niche overlap index is predicted to be smaller than those with low mobility. This supposition was largely supported by our results showing that Australian Wood Duck had the highest between‐year Schoener's D while Grey Teal had the lowest. In Victorian hunting fields, 40% of Australian Wood Duck recoveries were within 100 km of the banding site (Norman 1971). Only 3% were retrieved further than 500 km from the banding site. The low mobility of this species is reflected the high overlap of suitable habitat during dry and wet year (similarity index 0.692, the highest among the six species). By contrast, Grey Teal are known to be capable of traveling long distance in response to flooding events (Roshier et al. 2008) corresponding to the lowest similarity index, especially at inland NSW (0.436).

The comparison of the distribution between wet and dry years confirmed our third hypothesis for all species but Australian White Ibis. Classified as a large wader with low mobility, we expected the between‐year distribution overlap of Australian White Ibis to be high, similar to that of Australian Wood Duck. However, our results indicated that the similarity index was among the lowest (0.441 in inland NSW). The opportunistic nature of this species might explain the discrepancy. In the dry phase, a large population increase in urban areas was reported (Martin et al. 2010) scavenging at landfills and picnic grounds, consuming almost any relatively fresh food type (Shaw 1999).

Similarly, our fourth hypothesis was also largely supported in that the inland distribution overlap between different species was higher in the dry year except in two cases (i.e., Australian Wood Duck – Grey Teal and Masked Lapwing – Little Black Cormorant) indicating the prevailing poor condition drove the waterbirds to congregate in the remaining suitable habitats. By contrast, the similarity of between‐species distribution was ranked high to very high in both dry and wet years, and difference between wet and dry years was random due to two possible reasons. Firstly, the difference in NDVI variables was insufficient between dry and wet years within the coastal zone (Fig. 5) to allow discrimination, and secondly, the relatively small geographic size of suitable habitat in both wet and dry years might confound the discriminative power of Schoener's D.

### Changes in habitat suitability and implications for management

Reid et al. (2013) found the composition of waterbird assemblages in the Murray–Darling Basin (which overlaps significantly with our study area; cf. Fig. 1) differed according to ecosystem health of rivers (Davies et al. 2010), determined largely by the degree of hydro‐ecological change due to regulation and irrigation diversions. The colonially nesting waterbird guild (including Australian White Ibis) was associated with rivers in the north and west in moderate‐to‐good ecosystem health, indicating that the least altered rivers and their floodplains and wetlands provided the most suitable available habitat for this guild. High reporting rates of other species (with increased occurrence between 1984 and 2003, including Australian Wood Duck, Masked Lapwing, and Eurasian Coot), in the absence of a diversity of other species, aligned with rivers ranked poor or extremely poor.

The findings of Reid et al. (2013) indicate an association between habitat suitability for waterbirds and anthropogenic pressures on water resources of inland rivers in eastern Australia that are associated with reductions in frequency, duration, and extent of flooding of major wetlands (Sims et al. 2012). Anthropogenic changes in flow and flood regimes are compounded by the effects of drought, likely to become more frequent and prolonged under climate change (Leblanc et al. 2012; Van Dijk et al. 2013). In line with the study of Maclean et al. (2008), which found range shift of waders, our modeling demonstrated waterbird distribution ranges shifted from inland to coasts during drought and suggesting that coastal environments will become increasingly important in sustaining waterbird populations on the continent as more severe and frequent droughts were projected under future climate change scenarios.

In coastal regions, waterbird habitat is threatened by the phenomenon of “coastal squeeze” (Clausen and Clausen 2014), whereby loss of habitat due to anthropogenic pressures from urban and industrial development is compounded by effects on habitat quality and availability from increased frequency and severity of extreme tidal and storm surge events. Loss of habitat is likely to increase under sea level rise driven by climate change (Rogers et al. 2014). Given that these pressures apply to many important migratory waterbird sites worldwide, population changes at one site are likely to influence those at others (Hansen et al. 2015).

The design and use of environmental flows to restore elements of the flow regime of rivers represents an important management response to anthropogenic pressures on water resources (Arthington 2012; Acreman et al. 2014). Successful breeding by waterbirds is one of the most common objectives for the management of environmental flows (e.g., MDBA, 2014). There are thresholds for duration and magnitude of flooding flows which must be exceeded for breeding by colonially nesting waterbirds to commence (Arthur et al. 2012). Thresholds vary according to species and location. For regulated rivers and wetlands, management of breeding events requires a knowledge of the flow variables required to trigger breeding, as well as the duration and extent of inundation required to support food resources for the rearing of young (Leslie 2001; Kingsford and Auld 2005).

Long‐term monitoring of waterbird populations (Kingsford et al. 2013; Hansen et al. 2015) provides a basis for the identification of trends, patterns of variation, and, potentially, drivers of change for particular species at specific wetlands (Colloff et al. 2015). Distribution modeling, such as that reported herein, can help inform management decisions by determining how habitat availability varies between species with different habitat requirements, by highlighting changes in use by waterbird of wetlands that are have been historically breeding sites, and by identifying spatial and temporal patterns of refugia.

With over 50% of the suitable habitat for half of the studied species was located in coastal zones during drought, our study highlights the need to identify and preserve coastal drought refuges. Several decades ago, White (1987) expressed concern that most of the waterbird drought refuges in the New England Tablelands were being modified. Only recently has state‐wide monitoring of changes in extent and condition of wetlands commenced in New South Wales (Claus et al. 2011) and has been hampered by a lack of detailed inventory on wetland extent and distribution. Such monitoring will provide for a comprehensive assessment of wetland habitat availability, halt the tyranny of small decisions incrementally decreasing drought refuge for waterbirds, and provide a sound basis for the conservation and management of waterbirds into the future.

## Conflict of Interest

None declared.
